# Internationally trained nurses and host nurses’ perceptions of safety culture, work-life-balance, burnout, and job demand during workplace integration: a cross-sectional study

**DOI:** 10.1186/s12912-021-00581-8

**Published:** 2021-05-17

**Authors:** Catharina Roth, Sarah Berger, Katja Krug, Cornelia Mahler, Michel Wensing

**Affiliations:** 1 Department of General Practice and Health Services Research, Heidelberg University Hospital, Marsilius Arcades, West Tower, Im Neuenheimer Feld 130, 69120 Heidelberg, Germany; 2 Centre for Postgraduate Nursing Studies, University of Otago-Christchurch Campus, 2 Riccarton Ave, Christchurch, 9140 New Zealand; 3 Department of Nursing Science, University Hospital Tuebingen, Hoppe-Seyler-Str. 9, 72076 Tuebingen, Germany

**Keywords:** Migrant nurses, Host nurses, Workforce, Integration, Perceptions, Burnout, Germany

## Abstract

**Background:**

The shortage of qualified nurses is a problem of growing concern in many countries. Recruitment of internationally trained nurses has been used to address this shortage, but successful integration in the workplace is complex and resource intensive. For effective recruitment and retention, it is important to identify why nurses migrate and if their expectations are met to ensure their successful integration and promote a satisfying work climate for the entire nursing team. The aim of this study was to examine the perceptions of safety culture, work-life-balance, burnout and job demand of internationally trained nurses and associated host nurses in German hospitals.

**Methods:**

A multicentric, cross-sectional study was conducted with internationally trained nurses (*n* = 64) and host nurses (*n* = 103) employed at two university hospitals in the state of Baden-Wuerttemberg, Germany. An anonymous paper-based survey was conducted between August 2019 and April 2020. The questionnaire included sociodemographic questions, questions regarding factors related to migration, and the German version of the *Assessment of your work setting Safety, Communication, Operational Reliability, and Engagement* (SCORE) questionnaire. SCORE is divided into three sections: Safety Culture Domains (*six subscales*), Work-Life-Balance (*one subscale*), and Engagement Assessment Tool (*four subscales*).

**Results:**

Nurses who migrated to Germany were primarily seeking better working conditions, a higher standard of living, and professional development opportunities. Internationally trained nurses reported lower work-related burnout climate (Mean 55.4 (SD 22.5)) than host nurses (Mean 66.4 (SD 23.5)) but still at a moderately high degree *(Safety Culture Domains).* Host nurses indicated a higher workload (Mean 4.06 (SD 0.65)) (*Engagement Assessment Tool*) and a lower Work-Life-Balance (Mean 2.31 (SD 0.66)) *(Work-Life-Balance)* compared to nurses who trained abroad (Mean 3.67 (SD 0.81) and Mean 2.02 (SD 0.86), respectively). No differences were detected for the other subscales. The Safety Culture Domains and Engagement Assessment Tool showed room for improvement in both groups.

**Conclusion:**

The study suggest that the expectations migrant nurses had prior to migration may not be met and that in turn could have a negative impact on the integration process and their retention. With increasing recruitment of internationally trained nurses from within Europe but also overseas, it is crucial to identify factors that retain migrant nurses and assist integration.

**Trial registration:**

The study has been prospectively registered (27 June 2019) at the German Clinical Trial Register (DRKS00017465).

**Supplementary Information:**

The online version contains supplementary material available at 10.1186/s12912-021-00581-8.

## Background

The shortage of qualified nurses and understaffing is a problem of growing concern in many countries [1]. In recent years, the demand for qualified nurses has increased while the supply has been decreasing worldwide [2]. That means hospitals and other healthcare organisations are struggling to recruit and retain competent nurses. The reasons for this are multi-faced [3]. Insufficient staff, particularly in the nursing workforce, reduces performance of health systems including decreased responsiveness, lessening productivity and inequitable distribution of resources, which not only have negative impacts on quality and safety of patient care but also on the mental and physical health of nurses [4–8]. Health systems in Germany are increasingly confronted by problems arising from shortages in the nursing workforce [9, 10]. Inadequate numbers of nurses in direct patient care have been associated with poorer quality and safety outcome measures for patient morbidity and mortality [4–8]. Additionally, compounding effects of work-related stress due to factors such as high workload [5], inadequate numbers of support staff [11], work-related stress, lack of participation in decision making, and emotional exhaustion [11, 12] have been associated with both plans to leave the workforce or early exit by qualified nurses [13–17].

Factors associated with German nurses leaving the nursing workforce are mainly associated with workforce burnout, which is due to poor work environment including high workload, low recognition, poor teamwork, and staff shortages. This in turn is related to poor quality and safety in patient care [18, 19]. Additionally, poor leadership, limited growth opportunities, restricted autonomy as well as burnout contribute to the intention to leave the nursing profession [20]. Burnout is characterised by emotional exhaustion, depersonalisation and lower personal performance [21]. Around 45% of nurses working in hospitals in western countries are affected by burnout [22]. The interaction between the factors contributing to burnout and intention to leave is complex. For example, high workload contributes to a poor work environment and therefore workforce burnout. Limited growth opportunities and low remuneration have been associated with intention to leave the workforce, which in turn contributes to understaffing. Nursing shortages affects teamwork negatively, which has an impact on the learning environment, workforce burnout, and safety culture [8]. Safety culture focuses mainly on preventing medical errors and patient harm, but also on the mental and physical health of the medical team. Various factors such as effective communication, adequate staffing, appropriate work environment, collaborative teamwork, and a supportive leadership influence quality of safety culture [23]. Thus, poor safety culture is another factor that increases the risk of workforce burnout and turnover.

Germany has undertaken a range of country-level policy responses to nursing shortages, such as measures to improve retention and working conditions [2, 24], expand the recruitment base [24], target returners to the nursing workforce [2, 24], and more recently international recruitment of migrant health workers from the nursing profession [2, 24].

Migrant nurses typically move from rural to urban areas and from developing to industrialized nations [25]. As a high-income nation that is politically stable and has a strong economy, Germany can offer a range of incentives such as high quality of life and personal safety as well as opportunities for professional development and further qualification that are very attractive for migrant health workers [19]. Nurses who migrate to Germany are mainly from Austria, Eastern Europe, Southern Europe, and African countries [18]. Reasons for internationally trained nurses to migrate to Germany include difficult working and living conditions in their home countries, low status of nursing in home countries, minimal advanced training prospects as well as private and family reasons [18, 26]. Interestingly, some these reasons, particularly difficult working conditions, are consistent with the reasons German nurses have given for leaving their profession [18].

Recruitment of internationally trained nurses can be seen as a medium-term strategy to address nursing shortages. However, it is creating new challenges for healthcare organisations and the host nurses who supervise workplace entry of internationally trained nurses and support them to integrate into the German health workforce [27]. The focus of research on workplace integration of migrant nurses has been mainly on the challenges migrant nurses experience during their transition and orientation phase [28]. Challenges are, for example, the process of registration and complexity of registration requirements [29], adapting to a new nursing workplace (e.g. learning a new language or technical terminology), and to new work environment (e.g. different understanding of roles or responsibilities, or new practice contexts) [30, 31]. Other factors that have a negative impact on workplace integration are differences in nursing practice and cultural values, discrimination and racism, or delays in recognition of competencies, which can lead to deskilling and frustration [32, 33]. Successful workplace integration is a complex, costly [34] and time-consuming process not only for hospital management but also for internationally trained nurses [29, 35] and host nurses. For recruitment and retention purpose, it is important to not only focus on challenges during workplace integration but also to identify why nurses migrate to Germany and if their expectations are met to ensure their successful integration and promote a satisfying work climate for the entire nursing team [29].

As far as we know, no previous research has investigated the process of workplace integration of internationally trained nurses into the German nursing workforce. In particular no study, to our knowledge, has considered the importance of identifying why internationally trained nurses migrate to Germany and if their expectations are met. The aim of the study was to examine the nurses’ perceptions of safety culture, work-life-balance, burnout, and job demands during workplace integration among internationally trained nurses and associated host nurses.

## Methods

### Study design

A multicentric, cross-sectional study based on a paper-based survey was conducted between August 2019 and April 2020.

### Study setting

The setting for this study was the University Hospital Heidelberg and the Thorax Clinic Heidelberg. The University Hospital Heidelberg has fifty-seven specialized clinical departments (with 1.600 beds in total) and is a well-known medical centre in Europe. The Thorax Clinic Heidelberg (310 beds) is one of the largest lung care clinics in Germany and in Europe. Both hospitals provide tertiary level medical care. The two hospitals were selected, because they apply the strategy to actively recruit internationally trained nurses. Four more hospitals were invited to participate in this research but did not want to take part due to non-specific reasons (such as no interest in research or too time-consuming).

### Sampling and recruitment

A contact person was identified at each hospital as a key contact to inform the ward managers of eligible units within the two hospitals by the hospital management. Within these hospitals only wards that employed internationally trained nurses were selected. Psychiatric wards were excluded from the study due to differences in staffing and shift patterns. Ward managers were informed about the recruitment process and the purpose of the study by a member of the research team either during a meeting with all ward manager of eligible wards (Thorax Clinic Heidelberg) or by the key contact person (University Hospital Heidelberg). The meeting at the Thorax Clinic Heidelberg was organized by the key contact person. The ward managers then were responsible for distributing information resources to all potential study participants via the internal mail system and for explaining the purpose and nature of the project. The information resources included an information leaflet, the paper-based survey, and a reply envelope.

Two different, yet linked populations of nurses were eligible to take part in this study: (i) internationally trained nurses who migrated to Germany and (ii) host nurses (trained in Germany) who work with internationally trained nurses, and supported their integration into the German workforce in a hospital setting. The aim was to recruit a full census sample of internationally trained nurses and a full census sample of host nurses working with internationally trained nurses within each hospital.

### Inclusion and exclusion criteria

(i) Internationally trained nurses: to be eligible, participants had to have obtained a recognised qualification in nursing outside Germany and were working as a nurse in Germany, with or without a (full) registration to practice as a nurse in Germany and were over 18 years of age. Although nurses without a full registration to practice as a nurse in Germany are employed as nursing assistants until they obtained their registration, they were qualified nurses and were trained and were integrated into the nursing teams as a nurse equivalents under supervision.
(ii) Host nurses: to be eligible, participants had to have obtained a recognised qualification in nursing in Germany and to have worked on a ward where internationally trained nurses were part of the nursing team, and were over 18 years of age. No other inclusion criteria (e.g. being responsible for integration) were set.

Both, internationally trained nurses and host nurses, had to have an employment contract with either the University Hospital Heidelberg or the Thorax Clinic Heidelberg and internationally trained nurses had to have demonstrated written and spoken German language skills. Healthcare professionals other than nurses were excluded (e.g. physicians, physiotherapists), student nurses or internationally trained nurses and host nurses who did not consent to participate or who were not able to consent to participate. No minimum working hours were set as inclusion criteria.

### Data collection

Data was collected using an anonymous paper-based questionnaire. Host nurses who were eligible to participate in this study were identified based on the inclusion criteria and invited by their ward manager. Internationally trained nurses employed by one of the two recruiting hospitals that met inclusion criteria at the time of the study were invited to complete a questionnaire by their ward manager. All nurses received post via the internal mail system with an information leaflet explaining the purpose and the nature of the study, and a hard copy of the questionnaire. The information sheet included the contact details of a research team member in case participants wanted to discuss the study or had additional concerns or questions. Nurses were asked to return their questionnaires in sealed envelopes in a secure box on the hospital ward or to post it via the internal mail system. Mail reminders were sent to the nursing management of each hospital four weeks and six weeks after the initial distribution of the survey to increase respondents’ rate of return. Additionally, ward mangers of all included units were asked to promote the study during regular team meetings.

### Measures

The first part of the questionnaire contained standardised questions on, gender, age, marital status, children, work experience, and working hours per contract. The second part included questions regarding factors related to migration (e.g. length of residency, reasons for migration, and German language proficiency level based on the Common European Framework of Reference for Languages (CEFR)) [36]. Possible answer options were provided, no open-end questions were asked. These variables were used for analysis of demographic background, the factors related to migration, and for explanatory covariates in the regression analysis.

The third part of the written survey comprised the German version of the SCORE questionnaire: *Assessment of your work setting Safety, Communication, Operational Reliability, and Engagement* [37]. SCORE is divided into three sections: the Safety Culture Domains (*six subscales*), Work-Life-Balance (*one subscale*), and the Engagement Assessment Tool (*four subscales*).

Section 1: Safety Culture Domains measure patient safety culture and included the following six subscales: Learning environment (Improvement Readiness), Local Leadership, Burnout Climate and Personal Burnout, Teamwork Climate, and Safety Climate. The five point response scale ranged from 0 *strongly disagree* to 100 *strongly agree*, so that higher scores reflect higher safety culture [37]. The subscale “Learning Environment or Improvement Readiness” (*five items*), measures whether quality improvement was supported within a work setting through analysing strengths and weaknesses *(*e.g. *the learning environment in this work setting is protected by our local management)*. The subscale “Local Leadership” (*five items*), indicates to what extent leaders communicate with their employees and how available they are for their employees *(*e.g. *in this work setting local management is available at predictable times).* “Personal Burnout” (*five items*), measures emotional exhaustion *(*e.g. *I feel frustrated by my job).* “Burnout Climate” (*five items*), indicates the exhaustion climate or workforce burnout *(*e.g. *people in this work setting are burned out from their work).* Higher scores reflect higher levels of burnout. The subscale “Teamwork Climate” (*seven items*), shows whether interactions, such as collaboration or communication as well as the way conflicts are managed is effective *(*e.g. *dealing with difficult colleagues is consistently a challenging part of my job).* “Safety Climate” (*seven items*), measures to what extent patient safety regulations are actively applied, such as handling and learning from errors *(*e.g. *errors are handled appropriately in this work setting)* [37].

Section 2: The Work-Life-Balance section has a response scale from 1 (*Rarely or none of the time)* to 4 (*All of the time*). Lower scores reflect a better work-life-balance.

Section 3: The Engagement Assessment Tool consists of four subscales, Growth Opportunities, Workload, Participation in Decision Making, and Advancement. Each subscale uses the response scale of 1 (*strongly disagree*) to 5 (*strongly agree*), higher scores therefore indicate more positives norms for advancement, growth opportunities and inclusive decision-making but worse norms regarding workload.

Specific threshold values were based on the equivalent of *slightly agree* or *strongly agree* on the Likert-Scale used for the response options. Thus, mean scores above 50 indicated more positive perceptions towards Teamwork Climate, Learning Environment, Local Leadership and Safety Climate. Mean scores less than 75 or equal show that there is room for improvement in this areas. High levels of Personal or Workforce Burnout were defined as having mean scores above 50. Mean scores less than 75 or equal were considered as moderately high degree of burnout, means scores above 75 were considered as high burnout levels. Mean scores below 2.0 reflected fewer problems with Work-Life-Balance, the equivalent of *rarely or none of the time* and *some or a little of the time*. Mean scores above 3.0 indicated more positives norms for Advancement, Growth Opportunities and Participation in Decision Making but worse norms regarding Workload, the equivalent of *slightly agree* or *strongly agree*. Mean scores less than 4.0 or equal indicated that improvements in this area are needed.

The assessment SCORE was developed and available in English. The instrument was translated into German using a best practice approach translation-back translation method according to the ISPOR guidelines for translation [38, 39]. The original version showed good psychometric properties for each section (Section 1: between 0.82 and 0.96; Section 2: 0.83, Section 3: between 0.84 and 0.92) [40]. The Cronbach’s alpha for the translated version showed an acceptable internal consistency (Section 1: between 0.70–0.91; Section 2: 0.88; Section 3: between 0.80 and 0.90) (Table 5, supplementary material).

### Data analysis

Prior to analysis, all variables were checked for data entry errors and missing values. Descriptive statistics were used to calculate the means and standard deviations for continuous variables and frequencies and percentages for categorical variables. The Shapiro-Wilk-test of normality was used to determine whether the study sample was normally distributed. The standard technique to calculate mean scores for all 11 subscales of the SCORE was used [40]. Additionally, Levene’s test was used to assess equality of variances for the 11 mean scores of the subscales of the SCORE. Analysis of covariance (ANCOVA) was applied to identify significant difference in the mean scores of the 11 subscales of the SCORE between the two groups adjusted for gender and age.

Cohen’s D was calculated to determine the magnitude of the differences in the mean scores of the 11 subscales of the SCORE between the two groups to identify if relevant effects are found. Values between 0.2 or less are considered as small effect, values between 0.2 and 0.5 as a medium effect size, and values of 0.8 or larger as large effect [41].

Cronbach’s alpha was calculated to accessed the internal consistency for the German version of the score (Table 5, supplementary material). A subscale was considered as having a sufficiently internally consistent if Cronbach’s alpha was ≥ 0.70 [42].

Dummy coded variables were created for categorical variable (e.g. for age) in order to included them in the regression analysis. Bivariate linear regression analyses were applied to explore the impact of sociodemographic characteristics and factors related to migration on the 11 subscales of the SCORE. Sociodemographic characteristics or factors related to migration with significant effects in the bivariate regression were included in stepwise linear regression models adjusted for age and gender for both groups separately.

**Fig. 1:**
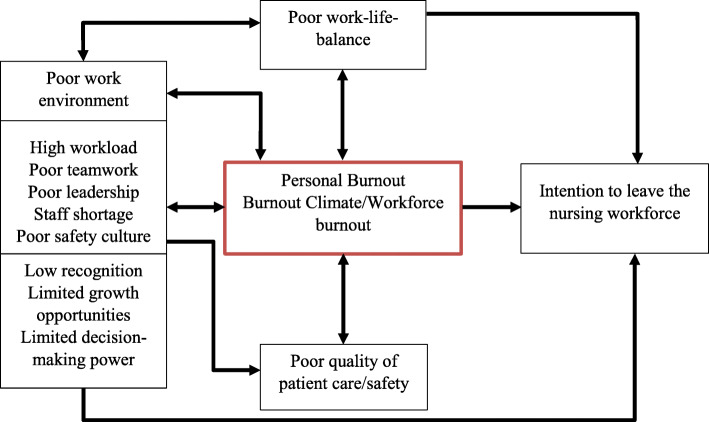
Tentative conceptual model to guide data-analysis (author’s diagram)

To guide data-analysis, a tentative conceptual model based on the literature described in the background section and on factors related to the intention to leave the nursing profession was developed (Fig. 1) [8, 18–22]. The conceptual framework was use as a heuristic tool rather than a theory that provided strong hypothesis. The framework suggests that factors which are associated with an increased risk for burnout or may cause burnout interact with each other and therefore contribute to the intention to leave the nursing workforce. All analyses were performed in IMB SPSS Statistics 25. Statistical significance was defined as *p* < 0.05 for all analysis.

### Ethical considerations

Ethical approval was obtained of the Medical Ethics Committee of the Medical Faculty of Heidelberg University (S-367/2019) prior to the start of the study. The staff council of both hospitals approved the study. Informed consent was obtained if participants completed the survey and posted it back to the research group. Research conducted in this study was performed in accordance with the Declaration of Helsinki.

## Results

Of all 264 internationally trained nurses who were identified as potential participants, 64 responded (response rate 24.24%). Out of a potential 400 host nurses who were invited to participate, 103 completed the questionnaire (response rate 25.75%). The majority of participants were from the University Hospital Heidelberg (89.1% internationally trained nurses, 68.9% host nurses). Since the University Hospital Heidelberg is larger (e.g. more beds and more employees) than the Thorax Clinic Heidelberg the different distribution among those facilities is representative.

### Description of the study population

The majority of internationally trained nurses were from Serbia (43.8%) or Bosnia-Herzegovina (42.2%). Most participating nurses identified as female (70.3% internationally trained nurses; 80.6% host nurses). Host nurses were distributed over all age categories, while internationally trained nurses tended to be younger (between 18 and 29 years (54.7%)). More than half of the host nurses indicated that they had more than five years work experience (54.4%), only a few stated they had less than one year of work experience (12.6%). The same was true for internationally trained nurses, almost half of them had more than five years professional experience (40.6%), however almost a third indicated that they had less than one year experience (26.6%). The majority of internationally trained nurses worked full-time (89.1%). Two thirds of the host nurses worked full-time (67.0%) (Table 1).

**Table 1:** Description of the study population *N* = 167 (100%) per study group

n (%)	Host nurses n = 103 (61.7)	Internationally trained nurses n = 64 (38.3)
**Centre**		
University Hospital Heidelberg	71 (68.9)	57 (89.1)
Thorax Clinic Heidelberg	32 (31.1)	7 (10.9)
**Country of Origin**		
Republic of Serbia	n/a	28 (43.8)
Bosnia-Herzegovina		27 (42.2)
Other		6 (9.4)
No answer		3 (4.7)
**Gender**		
Female	83 (80.6)	45 (70.3)
Male	18 (17.5)	16 (25.0)
Third	1 (1.0)	0
No answer	1 (1.0)	3 (4.7)
**Age group**		
18 to 29 years	28 (27.2)	35 (54.7)
30 to 40 years	30 (29.1)	18 (28.1)
40 to 50 years	19 (18.4)	7 (10.9)
over 50 years	24 (23.3)	1 (1.6)
No answer	2 (1.9)	3 (4.7)
**Marital Status**		
Single	26 (25.2)	29 (45.3)
Widowed	1 (1.0)	0
Separated	4 (3.9)	2 (3.1)
Divorced	8 (7.8)	0
Married/with partner	62 (60.2)	28 (43.8)
No answer	1 (1.0)	5 (7.8)
Other	1 (1.0)	0
**Children under the age of 18**		
yes	29 (28.2)	18 (28.1)
no	73 (70.9)	45 (70.3)
No answer	1 (1.0)	1 (1.6)
**Work experience as nurse**		
less than 1 year	13 (12.6)	17 (26.6)
between 1 and 3 years	16 (15.5)	15 (23.4)
between 3 and 5 years	14 (13.6)	5 (7.8)
more than 5 years	56 (54.4)	26 (40.6)
No answer	4 (3.9)	1 (1.6)
**Working hours by contract**		
100%	71 (68.9)	57 (89.1)
between 75 and 99%	21 (20.4)	0
between 50 and 74%	8 (7.8)	4 (6.3)
less than 50%	3 (2.9)	1 (1.6)
No answer	0	2 (3.1)

A range of reasons for migration to Germany were mentioned. Around a fifth of participants reported that they migrated to Germany for a higher standard of living (18.0%) and better working conditions (18.0%). A third stated as reasons for migration professional development (14.0%) and career opportunities (13.3%). Other nurses were looking for higher income (11.3%) and personal safety (11.3%). Only a few indicated other reasons. The majority of internationally trained nurses migrated alone (e.g. without family or friends) (82.8%) but via mediating organisation (82.8%). Internationally trained nurses stated that they had lived in Germany between 0 and 12 months (54.7%), between 12 and 24 months (34,4%), or for more than 24 months (7.8%). More than half of all internationally trained nurses participating in this study had achieved B2 German level (57.8%) on the Common European Framework of Reference for Languages (CEFR), which corresponds to the fourth level of a six-level scale (B1 and B2 indicate an *Independent User*, and C1 indicates a *Proficient User*) (Table 2).

**Table 2:** Migration-related aspects reported by internationally trained nurses

Reasons to migrate to Germany ^(a)^	n = 64 (100)
Better Working Conditions	27 (18.0)
Higher Standard of Living	27 (18.0)
Professional Development	21 (14.0)
Career Opportunities	20 (13.3)
Higher Income	17 (11.3)
Personal Safety	17 (11.3)
Opportunities for family members	10 (6.7)
Learning Opportunities	8 (5.3)
Better Future	2 (1.3)
No answer	1 (0.7)
**With whom did you migrate to Germany?**	
Alone	53 (82.8)
Family/Friends/Classmates	5 (7.8)
Other	2 (3.1)
No answer	4 (6.3)
**How did you migrate**?	
Mediated by an agency	53 (82.8)
Personal initiative	8 (12.5)
No answer	3 (4.7)
**Length of time living in Germany?**	
between 0 and 12 months	35 (54.7)
between 12 and 24 months	22 (34.4)
more than 24 months	5 (7.8)
No answer	2 (3.1)
**CEFR – German language proficiency**	
B1	17 (26.6)
B2	37 (57.8)
C1	5 (7.8)
C2	2 (3.1)
I don’t know	1 (1.6)
No answer	2 (3.1)

^(a)^ Multiple responses possible

### Differences of the means of the subscales of the SCORE between host nurses and internationally trained nurses

Out of 11 subscales of the SCORE, three showed a difference between the two samples of nurses.

### Burnout climate *(Section: Safety Culture Domains)*

There was a difference in the mean scores on the subscale for perceived Burnout Climate between internationally trained nurses (55.4 (22.5)) and host nurses (66.4 (23.5)); F(2, 157) = 3.65, *p* = 0.028) while adjusting for gender and age differences. These results suggest that internationally trained nurses perceived lower levels of Burnout Climate than host nurses but both groups evaluated the degree of emotional exhaustion climate moderately high (Table 3).

### Work-life-balance *(Section: Work-Life-Balance)*

ANCOVA for the Work-Life Balance subscale adjusted for gender and age differences showed that there was a difference between internationally trained nurses (2.02 (0.86)) and host nurses (2.31 (0.66)); F (2, 153) = 7.56, *p* = 0.001) which indicated that internationally trained nurse evaluated their Work-Life-Balance slightly better than host nurses but still perceived problems with the balance to some extent (Table 3).

### Workload *(Section: Engagement Assessment Tool)*

There was a difference between internationally trained nurses (3.67 (0.81)) and host nurses (4.06 (0.65)); F (2, 157) = 3.71, *p* = 0.027) for the subscale Workload adjusted for gender and age. These results suggest that internationally trained nurses perceived moderately lower levels of workload burden compared to host nurses but still high levels (Table 3).

**Table 3:** Results of the analysis of covariance (ANCOVA) on the 11 subscales of the SCORE questionnaire

	Host Nurses mean (SD)	Internationally trained Nurses mean (SD)	F	***p***-value	Cohen’s D
Safety Culture Domains					
Learning Environment ^(a)^	62.5 (18.5)	63.7 (12.9)	1.65	0.195	n/a
Teamwork Climate ^(a)^	54.5 (16.5)	55.2 (13.0)	1.82	0.166	n/a
Local Leadership ^(a)^	62.3 (22.2)	61.5 (15.5)	1.25	0.561	n/a
Safety Climate ^(a)^	57.1 (17.6)	57.2 (12.1)	2.29	0.425	n/a
Burnout Climate ^(b)^	66.4 (23.5)	55.4 (22.5)	3.65	0.028*	0.47
Personal Burnout ^(b)^	49.0 (25.9)	46.9 (23.1)	0.26	0.767	n/a
Work-Life-Balance					
Work-Life-Balance ^(c)^	2.31 (0.66)	2.02 (0.86)	7.46	0.001*	0.38
Engagement Assessment Tool					
Growth Opportunities ^(d)^	3.54 (0.76)	3.45 (0.66)	0.78	0.460	n/a
Workload ^(d)^	4.06 (0.65)	3.67 (0.81)	3.71	0.027*	0.53
Participation in Decision making ^(d)^	3.46 (0.67)	3.38 (0.50)	1.01	0.366	n/a
Advancement ^(d)^	3.04 (0.74)	3.21 (0.74)	1.68	0.189	n/a

^(a)^ *Positive perceptions were defined as having scale scores > 50;* ^(b)^ *Moderately high levels of emotional exhaustion were defined as having scales scores > 50 ≤ 75;* ^(c)^ *Mean scores ≤ 2.0 reflect fewer problems with work-life balance;* ^(d)^ Higher scores (≥3.0) indicated more positives norms for advancement, growth opportunities and inclusive decision-making but worse norms regarding workload; Statistical comparisons were based on the mean scores and the variation.; Models are adjusted for gender and age; *Statistical significance was defined as p < 0.05

Other differences among internationally trained nurses and host nurses were not significant.

### Teamwork climate and safety climate *(Section: Safety Culture Domain)*

The results of the Teamwork Climate subscale (55.2 (13.0) and 54.5 (16.5)) showed that the nursing teams tended to collaborate and communicate effectively and that conflicts were successfully resolved some extent. The mean scores of Safety Climate subcale (57.2 (12.1) and 57.1 (17.6)) indicated that patient safety measures were adopted to a moderate degree by the nursing teams (Table 3).

### Learning environment and local leadership *(Section: Safety Culture Domain)*

The results of the Learning Environment subscale showed that both groups indicated a learning infrastructure that supported their quality improvement efforts (63.7 (12.9) and 62.5 (18.5), respectively) to a moderate high degree. The same was true for the Local Leadership subscale, which showed that both groups indicated their management created an environment where they felt psychologically safe to a moderate high degree (63.7 (12.99) and 62.5 (18.5)) (Table 3).

### Growth opportunities, participation in decision making, and advancement *(Section: Engagement Assessment Tool)*

The mean scores of subscales Growth Opportunities, Participation in Decision Making, and Advancement were on average above 3.0 suggesting that the nurses felt like they were given the opportunity for professional and personal growth (3.45 (0.66) and 3.54 (0.76)), and were able to participate in decision making to a moderate extent (3.38 (0.50) and 3.46 (0.67)). It indicated that they perceived there was reasonable remuneration for their performance to some extent (3.21 (0.74)) and (3.04 (0.74)) (Table 3).

### Determinants of the subscales of the SCORE of internationally trained nurses and host nurses

Sociodemographic characteristics (Table 1) and factors related to migration (Table 2) were included in a bivariate linear regression analyses to identify their impact on the 11 subscales of the SCORE. Sociodemographic characteristics or factors related to the migration with significant effects were included in a stepwise linear regression model adjusted for age and gender (Table 4). Results of the bivariate regression analysis can be found in the appendix (Table 6, supplementary material).

**Table 4:**
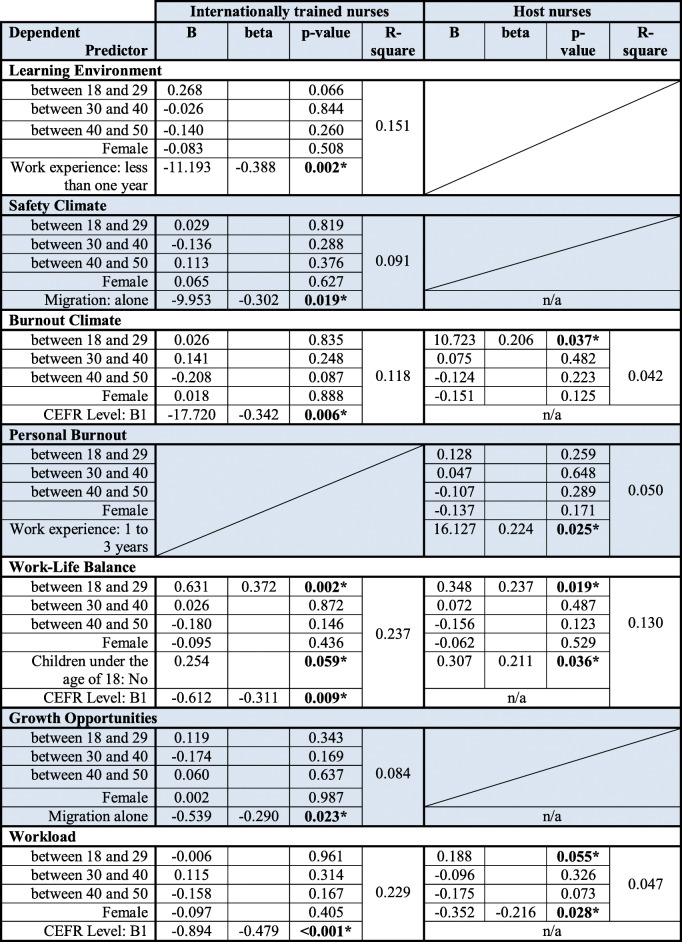
Results of the multiple stepwise regression analysis on the 11 subscales of SCORE per study group

*All models were adjusted for gender and age (Dummy coded); statistical significance was defined as p < 0.05; n/a (not applicable) indicates that either no significant effects were found or that the independent variable is for a certain study group not applicable

### Results of the stepwise linear regression model

Many of the presented associations between sociodemographic characteristics or factors related to migration and the mean scores of the 11 subscales of the SCORE were weak. However, 14 of the relationships were significant in the multiple regression analysis at a significance level at least *p* < 0.05, adjusted for gender and age indicating that there is evidence that a linear relationship exists.

### Learning environment *(Section: Safety Culture Domains)*

The multiple stepwise regression analysis showed that internationally trained nurses with less than one year professional experience tended to evaluate their Learning Environment as slightly less supportive (b = − 11.193, *p* = 0.002). No sociodemographic characteristics of host nurses were associated with Learning Environment.

### Safety climate *(Section: Safety Culture Domains)*

Internationally trained nurses who migrated to Germany alone evaluated the Safety Climate more positively compared to those who migrated with their family or with friends (b = − 9.953, *p* = 0.019). Results need to be interpreted with caution due to the small sample size (possible due to selection bias). No sociodemographic characteristics of host nurses showed an association with Safety Climate.

### Burnout climate and personal burnout *(Section: Safety Culture Domains)*

Host nurses between 18 and 29 years evaluated the degree of Burnout Climate higher compared to older host nurses (b = 10.723, *p* = 0.037). Internationally trained nurses with a level of language proficiency of B1 perceived higher levels of Burnout Climate (b = − 17.720, *p* = 0.006). Personal Burnout was associated with work experience in host nurses (b = 16.127, *p* = 0.025), indicating that nurses with more professional experience rather tended to feel burnt out.

### Work-life-balance *(Section: Work-Life-Balance)*

Host nurses without children under the age of 18 indicated less Work-Life-Balance problems (b = 0.207, *p* = 0.036) compared to those with children. Host nurses and internationally trained nurses older than 29 years tended to report more Work-Life-Balance problems (b = 0.348, *p* = 0.019, b = 0.631, *p* = 0.002, respectively). Internationally trained nurses with higher level of language proficiency (B2 or above) reported less Work-Life-Balance problems (b = − 0.612, *p* = 0.009).

### Growth opportunities *(Section: Engagement Assessment Tool)*

Internationally trained nurses who migrated alone tended to evaluate their Growth Opportunities better compared to nurses who migrated with their family (b = − 0.539, *p* = 0.023). Results need to be interpreted with caution due to the small sample size (possible due to selection bias).

### Workload *(Section: Engagement Assessment Tool)*

Host nurses who identified as male tended to perceive lower Workload levels compared host nurses who identified as female (b = − 0.352, *p* = 0.028). Internationally trained nurses with a higher level of language proficiency perceived lower Workload levels (b = − 0.894, *p* < 0.001) (Table 4).

## Discussion

Internationally trained nurses reported lower burnout climate than nurses trained in Germany. In contrast, host nurses indicated a higher perceived workload and a lower work-life-balance compared to nurses who trained abroad. The mean scores of the four subscales of the Engagement Assessment Tool and Teamwork and Safety Climate subscales showed room for improvement in both groups. A higher level of language proficiency was associated with a lower degree of burnout climate, a better work-life-balance, and lower perceived workload in internationally trained nurses.

Nursing workforce burnout is one of the main factors contributing to the decision of nurses to either leave their profession [12] or to migrate to another country [18]. In this study, both groups indicated a moderately low degree of personal burnout (degree below a mean score of 50) but a moderately high degree of burnout climate (degree above a mean score 50 and under 75). Inadequate working conditions are related to an increased risk of burnout and the intention to leave the nursing profession [5]. The moderately high degree of burnout climate among host nurses could contribute to an increased intention to leave in host nurses and further exacerbate perceptions of inadequate working conditions (e.g. due to staff shortage). In addition, Goh et al. [43] found out that work environment was associated with intention to leave of migrant nurses who migrated from East Asia and India to Singapore. This may also apply to the migrant nurses in this study, particularly since the results of this study showed that better working conditions were one of the main reasons for internationally trained nurse to move to Germany. However, cultural differences and beliefs need to be considered when comparing groups of migrant nurses. In addition, half of the migrant nurses of this study reported that they had lived in Germany for less than a year and were probably working on the ward for a shorter time. Burnout usually occurs in those who have been exposed to excessive stress at work over a period of time [44]. This needs to be considered when interpreting the results regarding burnout and work-life-balance in internationally trained nurses.

Research on burnout in nursing shows that adverse job characteristics, particularly high workload and staffing level is associated with a higher risk of burnout [45] and therefore intention to leave the profession. Host nurses in this study indicated a higher perceived workload and higher degrees of personal and workforce burnout. In everyday hospital practice host nurses may have to take more responsibility for patient care due to inadequate staffing and have to supervise their internationally trained colleagues during orientation and workplace integration. This may increase their perceived workload and could therefore be one factor, which contributed to higher degrees of personal and workforce burnout [45]. On the other hand, the orientation phase (e.g. additional theoretical training, language courses, adaption to a new work environment) could influence how internationally trained nurses perceive their workload [29]. Eliminating factors that contribute to inadequate working conditions and therefore increased risks of workforce burnout is critical for the retention of both migrant nurses and host nurses.

Internationally trained nurses in our study were mainly motivated by working conditions and higher standard of living but also by professional development opportunities. These results are consistent with those by Kishi et al. [46], Buchen et al. [26], and Zander et al. [18]. Winkelmann-Gleed et al. [47] on the other hand found out that some internationally trained nurses who migrated to the United Kingdom were denied opportunities to further professional development and only a few were promoted after they adjusted to their host country. Reasons for not offering opportunities to further professional development are, for example, length of time in the current position and the expression of migrant nurses regarding advancement opportunities and promotion [47]. In our study, migrant nurses tended to evaluate the possibility for professional growth and advancement opportunities as moderate. Offering additional training and development opportunities such as skill-based training programmes or workshops, and seminars to migrant nurses may contribute to a successful workplace integration. Furthermore, around half of the respondents in this study reported to having work experience between three and five years or more. This indicated that these nurses possibly had a competence level above a novice/beginner and would be considered as competent, proficient, or possibly experts in the country where they obtained their nursing qualification [48, 49]. The competence level of migrant nurses is too often overlooked given migrant nurses often come from highly skilled nursing positions or have several years of professional experience before migrating [33, 47, 50]. Deskilling can cause frustration and low job satisfaction [33, 47], which in turn increases intention to leave. Recognizing clinical skills and capabilities, and offering migrant nurses’ opportunities to further professional development, and providing career advancement opportunities may positively influence the integration process.

Eriksson et al. [51] found that internationally trained nurses who migrated to Sweden mainly evaluated the support they received from their line managers during integration as positive [51]. However, some migrant nurses stated that the lack of supportive management and feedback created feelings of uncertainty about work efficiency and caused feelings of being take advantages of by management [51]. Another study found that migrant nurses did not know who they should ask if they had question and that they experienced a lack of support by supervisors [52]. These results are in part consistent with the findings of this study. Migrant nurses in this study evaluated the degree of support they received from their nursing management as moderately high. Lack of support and an ineffective working relationship between the line manager and the nurse has been associated with intention to leave [53]. In addition, research shows that internationally trained nurses feel like outsiders within the nursing team and that they have to cope with loneliness and social isolation [35, 52]. Lack of support from peers fostered feelings of resentment and frustration [52] and feelings of being lost (e.g. not knowing who to ask or where to go) were common [52]. The results of this study showed that teamwork climate was evaluated moderate by both groups indicating that the nurses felt like they were working effectively as a team and supported each other to some extent. Research suggests that migrant nurses benefit from a strong support system, which can be found within the nursing team [34] or from a mentor system [54]. Despite support from host nurses, social groups and support from other migrant nurses decreased stress [52]. The majority of migrant nurses in this study stated that they migrated to Germany without family or friends. Thus, the support from fellow migrant nurses was probably not available, which in turn highlights the importance of a strong peer support system.

In this study, migrant nurses with higher levels of language proficiency indicated a lower perceived workload. One explanation could be that language barriers during patient care or teamwork decrease for nurses with proficient German language, which may have a positive impact on the degree of perceived workload. Although migrant nurses evaluated their perceived workload lower compared to their host nurses, the mean score was still moderately high. This finding aligns with those by Blythe et al. [29] and Takeno et al. [55]. Migrant nurses reported not being accustomed doing total patient care and were overwhelmed by paperwork [29, 55]. Although most migrant nurses adjusted to the workload [55], Blythe et al. [29] found out that some left the nursing profession due to the pressure experienced during the workplace integration. Thus, identifying factors that place additional burden on migrant nurses during their transition and addressing them may influence the integration process positively. High workload during transition increases work-related stress and may have a negative impact on the integration process. Eliminating factors that contribute to work-related stress should be of a priority for hospital managers to retain not only migrant nurses but also host nurses. Targeted interventions to address workforce burnout in migrant nurses and host nurses and factors contributing to emotional exhaustion may vary a cross settings and have to be considered.

In most parts of Germany the language level proficiency B2 is required to receive full registration to practice in Germany [56]. The results of this study show that lower levels of language proficiency predicted higher degrees of burnout climate, a higher perceived workload, and a lower work-life-balance. Thus, language proficiency seems to be an essential factor for internationally trained nurses and may has a negative impact on the integration process. This finding aligns with those by Adams et al. [57]. According to their findings, language and communication difficulties are one barrier that has a negative impact on the integration of nurses into the host country. Communication with patients and co-workers, including physicians and other healthcare professionals, is a core competence in nursing. Miscommunication and communication errors are associated with lower quality of patient care and safety [58]. Additionally, inacceptable standard of nursing care has an direct impact on the mental health of nurses and therefore increased risk of burnout [22].

In this study most, migrant nurses have been in Germany relatively short (less than one year). According to Adams et al. [57] and Habermann et al. [59] migrant nurses need on average 10 years to adjust completely to their new workplace. This indicates that migrant nurses in this study are only at the beginning of their integration process. For retention purpose it is important to consider that challenges during the integration process can arise after orientation phase. Thus, supporting migrant nurses not only during their orientation phase but also in the long run is crucial for the integration process to be successful.

### Limitations

This study has some limitations that may affect interpretation of the results. The cross-sectional design allows identifying association, but casual interference cannot be made. Thus, the results have to be interpreted with caution. All variables were assessed by self-reports; thus, results might be biased by common method variance, self-report, selection, or social desirability bias. Additionally, nurses filled in the survey while on duty, therefore responses could be influenced by events on the day or workload. Hence, the survey should be repeated to evaluate whether the results differ in terms of day and shift. The target population were migrant and host nurses employed at a university hospital generalising results beyond this study sample may be problematic. The University Hospital Heidelberg is actively recruiting nurses from the Balkan countries. Hence, results may not be generalizable to migrant nurses from other source countries. Future studies should be undertaken in different hospital settings and with migrant nurses from different source countries. No difference between setting e.g. intensive care unit or a normal ward was made. Hence, it is not possible to evaluate if the work setting has an impact on the results. Further research needs to be done on specific work settings to determine any potential impact on the results. The German language proficiency of migrant nurses may have had an impact on the proper understanding of the questionnaire. However, language proficiency levels equal B1 or above indicates that the person is able to understand common expressions. Despite repeated reminders to increase response rate e.g. promoting the study during nursing manager meetings and email reminder, only a low number of participants was reached thus results must be potential biased by non-response bias. Nurses from both groups with more difficulties may have not completed the questionnaire, which may have had an impact on results e.g. the true burnout rates (underestimation). Due to the small sample size results need to be confirmed in further prospectively target studies, particularly regards the results on gender and migration alone or with family/friends. The method of this study only offers quantitative data it is may be useful to include qualitative data to verify the results.

## Conclusion

Despite the limitations, the results of this study show that nurses migrating to Germany were mainly seeking better working conditions, a higher standard of living, and professional development opportunities. Nevertheless, they evaluated their workload as moderately high and indicated a moderately high degree of workforce burnout. In addition, growth opportunities and learning environment showed room for improvement. The same was true for their associated host nurses. This implies that the expectations migrant nurses had prior to migration may have not been met.

### Implications for practice

Factors that may promote workplace integration and retention of internationally trained nurses and host nurses included an adequate working environment, collaborative teamwork, a supportive management, career advancement opportunities, and a desirable work-life-balance. Interventions which reduce workload and improve work environment such as training programs which aim at improving supervision skills, peer mentoring programs, or teambuilding workshops, and therefor reduces the risk of burnout may contributes to a desirable work-life-balance and decreases the intention to leave particularly in host nurses. In addition, the implementation of such interventions, may reduce additional workload and thus risk of burnout in host nurses and therefore may contribute to the success of the integration process. Supporting migrant nurses to acquire higher levels of language proficiency via language courses focusing not only on everyday language but on medical language and local specifics should be of great interest for hospital management for retention purpose. Interventions which improve teamwork climate such as team building activities or joint activities may have a positive effect on the integration of migrant nurses in the nursing team and on the overall quality of teamwork. Finally, the relationship between migrant nurses, host nurses and their line mangers should be fostered, and a collaborative teamwork climate should be promoted to support the integration process, decrease risk of burnout, and enhance a desirable work-life-climate.

## Supplementary Information

**Additional file 1.**

## Data Availability

The dataset that was generated and analysed during the study will not be made publicly available due to German data protection law but may be made available by the corresponding author on reasonable request.
