# Supplementary material for: Internationally trained nurses and host nurses’ perceptions of safety culture, work-life-balance, burnout, and job demand during workplace integration: a cross-sectional study
Source: BMC Nurs. 2021 May 17;20:77. doi: 10.1186/s12912-021-00581-8 (PMC8127287; doi:10.1186/s12912-021-00581-8)
Supplement: Supplementary file 1 — Additional file 1. [file 12912_2021_581_MOESM1_ESM.docx]

**Internationally trained nurses and host nurses’ perceptions of safety culture, work-life-balance, burnout, and job demand during workplace integration: a cross-sectional study**

Catharina Roth^1^, Sarah Berger^2^, Katja Krug^1^, Cornelia Mahler^3^, Michel Wensing^1^

1 Heidelberg University Hospital, Department of General Practice and Health Services Research, Marsilius Arcades, West Tower, Im Neuenheimer Feld 130, 69120 Heidelberg, Germany

2 Centre for Postgraduate Nursing Studies, University of Otago-Christchurch Campus, 2 Riccarton Ave, Christchurch 9140, New Zealand

3 University HospitalTuebingen, Department of Nursing Science, Hoppe-Seyler-Str. 9 , 72076 Tuebingen, Germany

| Catharina Roth B.Sc, MPH  **Corresponding author** | Dept. of General Practice and Health Services Research,  University Hospital of Heidelberg, Marsilius Arkaden, Turm West  Im Neuenheimer Feld 130.3  69120 Heidelberg  Germany  Phone: +49 6221 / 56 35736  Email: catharina.roth@med.uni-heidelberg.de |
| --- | --- |
| Dr. Sarah Berger | Centre for Postgraduate Nursing Studies  University of Otago-Christchurch Campus  2 Riccarton Ave  Christchurch 8140  New Zealand  Email: sarah.berger@cdhb.health.nz |
| Dr. Katja Krug | Dept. of General Practice and Health Services Research,  University Hospital of Heidelberg, Marsilius Arkaden, Turm West  Im Neuenheimer Feld 130.3  69120 Heidelberg  Germany  E-Mail: katja.krug@med.uni-heidelberg.de |
| Prof. Dr. Cornelia Mahler | Dept. of Nursing Science  University Hospital Tuebingen  Hoppe-Seyler-Str. 9  72076 Tuebingen  Germany  Email: cornelia.mahler@med.uni-tuebingen.de |
| Prof. Dr. Michel Wensing | Dept. of General Practice and Health Services Research, University Hospital of Heidelberg, Marsilius-Arkaden, Turm West  Im Neuenheimer Feld 130.3  69120 Heidelberg  Germany  Email: michel.wensing@med.uni-heidelberg.de |

## Supplementary material

Table 5: Results of the Reliability Analysis of the German version of the SCORE

|  | **N** | **Number of items** | **Answering categories** | **Scale construction** | **Cronbach’s alpha** |
| --- | --- | --- | --- | --- | --- |
| Safety Culture and Well-being Assessment Tool | | | | | |
| Learning Environment | 154 | 5 | 5 | Average | 0.85 |
| Teamwork Climate | 100 | 7 | 5 | Average | 0.70 |
| Local Leadership | 159 | 5 | 5 | Average | 0.86 |
| Safety Climate | 136 | 7 | 5 | Average | 0.80 |
| Burnout Climate | 158 | 5 | 5 | Average | 0.91 |
| Personal Burnout | 158 | 5 | 5 | Average | 0.89 |
| Work-Life-Balance | | | | | |
| Work-Life-Balance | 109 | 8 | 4 | Average | 0.88 |
| Engagement Assessment Tool | | | | | |
| Growth Opportunities | 153 | 6 | 5 | Average | 0.85 |
| Workload | 163 | 5 | 5 | Average | 0.90 |
| Participation in Decision making | 153 | 6 | 5 | Average | 0.80 |
| Advancement | 150 | 7 | 5 | Average | 0.83 |

Table 6: Results of the bivariate regression analysis

| **Dependent**  **Predictor** | **Internationally trained nurses** | | **Host nurses** | |
| --- | --- | --- | --- | --- |
|  | **b** | **p-value** | **b** | **p-value** |
| **Learning Environment** | | | | |
| Work experience (0 – 1 year) | -10.07 | **0.011*** |  | |
| Work experience (1 – 3 years) | 1.83 | 0.662 |  |  |
| Work experience (3 – 5 years) | 4.90 | 0.416 |  |  |
| Reference Work experience (more than 5 years) | | | | |
| Age (18 – 29 years) |  | | -12.63 | **0.012*** |
| Age (30 – 40 years) |  |  | -2.55 | **0.039*** |
| Age (40 – 50 years) |  |  | -5.00 | 0.365 |
| Reference Age (over 50 years) | | | | |
| **Teamwork Climate** | n/a | | | |
| **Local Leadership** | | | | |
| Work experience (0 – 1 year) | -7.55 | 0.126 |  | |
| Work experience (1 – 3 years) | -0.48 | 0.928 |  |  |
| Work experience (3 – 5 years) | -15.90 | **0.041*** |  |  |
| Reference Work experience (more than 5 years) | | | | |
| Age (18 – 29 years) |  | | -14.61 | **0.016*** |
| Age (30 – 40 years) |  |  | -3.09 | **0.038*** |
| Age (40 – 50 years) |  |  | -4.55 | 0.494 |
| Reference Age (over 50 years) | | | | |
| **Safety Climate** | | | | |
| Age (18 – 29 years) |  | | -11.28 | **0.017*** |
| Age (30 – 40 years) |  |  | -2.77 | **0.017*** |
| Age (40 – 50 years) |  |  | -5.06 | 0.329 |
| Reference Age (over 50 years) | | | | |
| Migration alone | -9.953 | **0.019*** | n/a | |
| **Burnout Climate** | | | | |
| CEFR Level B1 | -17.720 | **0.006*** | n/a | |
| Work experience (0 – 1 year) | 8.824 | 0.196 | 7.44 | 0.295 |
| Work experience (1 – 3 years) | 6.731 | 0.364 | 13.54 | **0.039*** |
| Work experience (3 – 5 years) | 24.000 | **0.027*** | 7.32 | 0.287 |
| Reference Work experience (more than 5 years) | | | | |
| **Personal Burnout** | | | | |
| Work experience (0 – 1 year) | 12.99 | 0.062 | 12.29 | 0.122 |
| Work experience (1 – 3 years) | -2.31 | 0.758 | 20.04 | **0.006*** |
| Work experience (3 – 5 years) | 20.69 | **0.058*** | 14.247 | 0.065 |
| Reference Work experience (more than 5 years) | | | | |
| **Work-Life-Balance** | | | | |
| Age (18 – 29 years) | -0.037 | 0.928 | 0.333 | **0.057*** |
| Age (30 – 40 years) | -0.168 | 0.123 | -0.011 | 0.790 |
| Age (40 – 50 years) | -1.124 | **0.029*** | -0.355 | 0.071 |
| Reference Age (over 50 years) | | | | |
| Work experience (0 – 1 year) | 0.703 | **0.006*** | -0.026 | 0.196 |
| Work experience (1- 3 years) | 0.781 | **0.005*** | 0.518 | **0.005*** |
| Work experience (3 – 5 years) | 0.643 | 0.102 | 0.334 | 0.092 |
| Reference Work experience (more than 5 years) | | | |  |
| Not having children under the Age of 18 | 0.558 | **0.018*** | 0.410 | **0.004*** |
| CEFR Level B1 | -0.618 | **0.014*** | n/a | |
| **Growth Opportunities** | | | | |
| Age (18 – 29 years) |  | | -0.405 | **0.040*** |
| Age (30 – 40 years) |  |  | -0.067 | 0.167 |
| Age (40 – 50 years) |  |  | 0.002 | 0.992 |
| Reference Age (over 50 years) | | | | |
| Work experience (0 – 1 year) |  | | -0.129 | 0.555 |
| Work experience (1 – 3 years) |  |  | -0.417 | **0.040*** |
| Work experience (3 – 5 years) |  |  | -0.438 | **0.041*** |
| Reference Work experience (more than 5 years) | | | | |
| Migration without family/friends | -0.539 | **0.023*** | n/a | |
| **Workload** | | | | |
| CEFR Level B1 | -0.894 | **>0.001*** |  | |
| Period lived in Germany (0 – 1 year) | -0.785 | 0.013 |  |  |
| Period lived in Germany (1 – 2 year) | -0.134 | 0.679 |  |  |
| Reference Period lived in Germany ( 2 years or longer) | | |  |  |
| Gender Female | n/a | | -0.353 | **0.028*** |
| **Participation in Decision making** | n/a | | | |
| **Advancement** | n/a | | | |

** Statistical significance was defined as p<0.05; n/a (not applicable) indicates that either no significant effects were found or that the independent variable is for a certain study group not applicable*
